# Supplementary material for: Quantification of Fluid Volume and Distribution in the Paediatric Colon via Magnetic Resonance Imaging
Source: Pharmaceutics. 2021 Oct 19;13(10):1729. doi: 10.3390/pharmaceutics13101729 (PMC8540766; doi:10.3390/pharmaceutics13101729)
Supplement: Supplementary file 1 [file pharmaceutics-13-01729-s001.zip › pharmaceutics-1393638-supplementary.pdf]

pharmaceutics

Supplementary Materials: Quantification of Fluid Volume and Distribution in the Paediatric Colon via Magnetic Resonance Imaging

Jan Goelen , Benoni Alexander , Haren Eranga Wijesinghe , Emily Evans , Gopal Pawar , Richard D. Horniblow and Hannah K. Batchelor

Table S1. Data on participants demographics and MRI set-up.

ID	Age	Age (years)	Age group	Weight (kg)	Sex (Male (M) or Female (F))	Fed state	Hospital	Echo Time TE (ms)	Repetition time TR (ms)	Z-axis (cm)
2	2 w	0.04	newborn	N/A	F	fasted	BCH	241	2660.65	0.09
3	5 m	0.42	newborn	6.8	F	fasted	BCH	87	3200	0.55
4	5 m	0.42	newborn	7.1	F	fasted	BCH	241	4008.18	0.09
5	8 m	0.67	newborn	8.8	F	fasted	BCH	117	1400	0.16
6	12 m	1.00	newborn	9.7	F	fasted	BCH	241	3012.22	0.09
7	1 w	0.02	newborn	2.88	F	fasted	BCH	241	3012.22	0.09
8	1 y 10 m	1.83	newborn	11	M	fasted	BCH	221	2943.23	0.09
9	18 m	1.50	newborn	11	F	fasted	BCH	241	2844.65	0.09
10	12 m	1.00	newborn	7.2	M	fasted	BCH	704	4356.24	0.09
12	2 y 2 m	2.17	pre-school	10	F	fasted	BCH	240	2960.74	0.09
13	3 y 2 m	3.17	pre-school	16.3	F	fasted	BCH	241	3021.27	0.09
14	2 y 1 m	2.08	pre-school	12.2	F	fasted	BCH	241	3010.72	0.09
15	3 y 7 m	3.58	pre-school	12	M	fasted	BCH	241	2907.49	0.09
16	6 y 8 m	6.67	school-age	22.1	F	fasted	BCH	241	3161.32	0.09
18	5 y	5.00	pre-school	12.8	M	fasted	BCH	87	2200	0.55
19	4 y 5 m	4.42	pre-school	16.9	F	fasted	BCH	241	3011.71	0.09
20	6 y 2 m	6.17	school-age	N/A	F	fasted	BCH	241	2747.37	0.09

21	8 y 3 m	8.25	school-age	15.9	M	fasted	BCH	241	2900.01	0.09
22	9 y 1 m	9.08	school-age	39.1	F	fasted	BCH	87	3200	0.55
23	8 y 2 m	8.17	school-age	35.3	M	fasted	BCH	241	3022.19	0.09
24	9 y 8 m	9.67	school-age	28.7	F	fasted	BCH	241	3466.53	0.09
25	15 y	15.00	adolescents	N/A	M	fasted	BCH	242	3987.78	0.09
30	12	12.00	adolescents	33.5	F	fed	UHCW	1.8	4.822	0.6995
31	13	13.00	adolescents	52.7	M	fed	UHCW	1.896	5.695	0.498
32	12	12.00	adolescents	33.5	F	fed	UHCW	1.8	4.822	0.6995
34	15	15.00	adolescents	55	F	fed	UHCW	1.896	5.661	0.4997
35	16	16.00	adolescents	59	F	fed	UHCW	1.896	5.844	0.498
36	15	15.00	adolescents	65	F	fed	UHCW	1.896	5.604	0.5
37	15	15.00	adolescents	94	F	fed	UHCW	1.896	5.604	0.5
38	10	10.00	school-age	32.6	F	fed	UHCW	2.1	652.77	0.2998
39	11	11.00	school-age	28	M	fed	UHCW	2.1	652.77	0.3
40	13	13.00	adolescents	65	F	fed	UHCW	2.13	623.86	0.5
41	14	14.00	adolescents	54	F	fed	UHCW	1.72	4.253	0.7
42	15	15.00	adolescents	58	F	fed	UHCW	1.896	5.604	0.5
43	16	16.00	adolescents	54.4	M	fed	UHCW	1.9	5.716	0.4994
44	16	16.00	adolescents	65	F	fed	UHCW	1.9	5.764	0.4982
45	16	16.00	adolescents	60	F	fed	UHCW	1.896	5.604	0.5
46	16	16.00	adolescents	57	M	fed	UHCW	2.02	4.598	0.5988
47	16	16.00	adolescents	56	M	fed	UHCW	1.724	4.635	0.628
48	16	16.00	adolescents	102	M	fed	UHCW	1.888	5.519	0.4983
49	3	3.00	pre-school	N/A	N/A	fasted	BCH	241	3012.72	0.09
50	3	3.00	pre-school	N/A	N/A	fasted	BCH	241	2903	0.09

51	3	3.00	pre-school	N/A	N/A	fasted	BCH	221	3011.76	0.09
52	3	3.00	pre-school	N/A	N/A	fasted	BCH	241	3012.22	0.09
53	5	5.00	pre-school	N/A	N/A	fasted	BCH	241	4016.56	0.09
54	5	5.00	pre-school	N/A	N/A	fasted	BCH	241	3168.3	0.09

Table S2. Data on used threshold values, fluid volumes via both protocols and number of pockets in the total and segments of the colon per participant.

ID	Total colonic fluid volume via 2D protocol (mL)	Total colonic fluid volume via 3D protocol (mL)	Total Number of fluid pockets	Number of fluid pockets in ascending colon	Number of fluid pockets in transverse colon	Number of fluid pockets in descending colon	Threshold value	Fluid volume in ascending colon via 2D protocol (mL)	Fluid volume in transverse colon via 2D protocol (mL)	Fluid volume in descending colon via 2D protocol (mL)
2	0	0	0	0	0	0	292.94	0	0	0
3	67.998	69.0434	16	16	0	0	474.7	41.603	6.055	20.34
4	0.0046	N/A	N/A	N/A	N/A	N/A	334	0.0046	0	0
5	0.6755	0.817	19	19	0	0	450.91	0.6755	0	0
6	0.9164	1.0537	21	17	4	0	320.56	0.3458	0.1744	0.3962
7	0	0	0	0	0	0	248.97	0	0	0
8	0.2306	0.2962	19	19	0	0	316.78	0.2306	0	0
9	19.092	N/A	N/A	N/A	N/A	N/A	259.36	5.1182	13.964	0.0093
10	0	0	0	0	0	0	336.07	0	0	0
12	0.3219	0.3474	11	10	1	0	339.9	0.2905	0.0058	0.0257
13	0	0	0	0	0	0	302.92	0	0	0
14	0	0	0	0	0	0	396.7	0	0	0
15	0.0929	0.1196	5	5	0	0	253.81	0.0923	0.0005	0
16	19.835	19.8487	47	46	0	1	217.96	19.719	0	0.1161
18	131.49	249.602	1	1	1	1	262.35	81.123	12.423	37.943
19	2.6663	2.5484	35	31	4	0	186.71	2.5183	0.0281	0.12
20	22.226	N/A	N/A	N/A	N/A	N/A	167.42	19.704	0.6523	1.8694
21	0.3236	0.3515	32	32	0	0	355.95	0.1415	0.1822	0
22	0.4065	0.498	8	8	0	0	416.81	0	0.3966	0.0099
23	14.164	14.1689	34	34	0	0	142.79	12.436	1.4667	0.2614
24	62.872	44.942	19	13	6	0	296.71	61.925	0.9086	0.0392
25	0.0037	0.004	1	1	0	0	335.03	0	0.0037	0
30	14.238	14.2883	23	20	3	0	1526	10.153	4.0535	0.0322
31	167.47	N/A	N/A	N/A	N/A	N/A	1092.5	102.3	51.767	13.399
32	6.1234	N/A	N/A	N/A	N/A	N/A	1717.3	5.5002	0.6233	0
34	152.94	139.55	19	19	0	0	1808	75.205	56.867	20.868
35	60.011	65.7035	65	53	12	0	1425.6	42.458	14.839	2.7141
36	11.22	13.0425	26	26	0	0	1672.1	11.22	0	0
37	0	0	0	0	0	0	1526.7	0	0	0
38	92.208	86.2179	35	32	3	0	336.85	84.962	7.2465	0
39	0.0873	0.0842	2	2	0	0	227.13	0	0	0.0873

40	0.026	0.0257	8	8	0	0	229.6	0	0	0.026
41	2.0748	2.211	5	5	0	0	1462.8	2.0748	0	0
42	0	0	0	0	0	0	1592	0	0	0
43	0.5303	0	0	0	0	0	1793.1	0.5303	0	0
44	43.198	43.2104	13	13	0	0	1165.4	43.185	0.0055	0.007
45	0	0	0	0	0	0	2136.1	0	0	0
46	0	0	0	0	0	0	939.84	0	0	0
47	6.1601	7.6259	1	1	0	0	1360.7	6.1601	0	0
48	0.5775	0.1449	7	7	0	0	1775	0.5775	0	0
49	15.239	26.8771	24	24	0	0	223.91	14.977	0.0049	0.2563
50	22.917	41.5957	13	13	0	0	217.63	22.917	0	0
51	0.2453	0.2919	24	24	0	0	271.35	0.082	0.1346	0.0287
52	0.4325	N/A	N/A	N/A	N/A	N/A	216.05	0.1685	0.2582	0.0058
53	75.708	81.3068	17	10	7	0	197	73.249	0.3104	2.1482
54	19.414	18.5813	71	71	0	0	168.84	14.384	1.4831	3.5474

Table S3. correlation of threshold with volumes and number of pockets.

Correlation of threshold with:	Pearson's correlation coefficient	P	N
Fluid volume in the total colon	0.085	0.573	46
Fluid volume in the ascending colon	0.015	0.924	46
Fluid volume in the transverse colon	0.253	0.090	46
Fluid volume in the descending colon	0.030	0.846	46
Number of pockets	-0.169	0.297	40

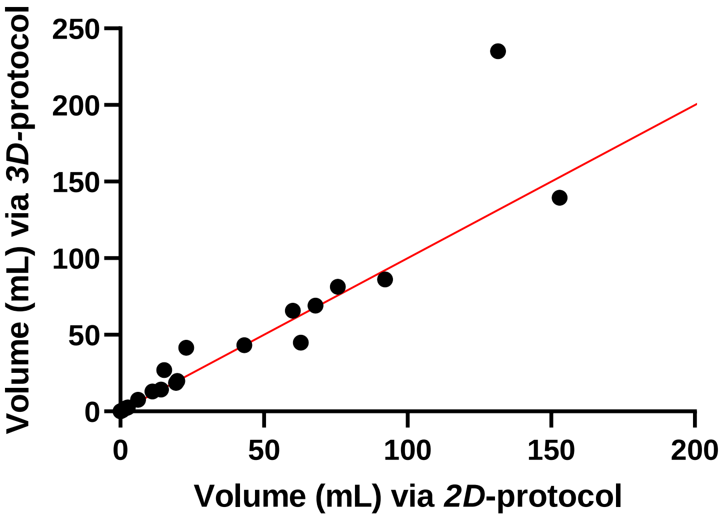

Figure S1. correlation of the fluid volumes in the total colon on the same datasets between the *2D*- and *3D*-protocol (Pearson's correlation coefficient 0.939, $p < 0.05$, $n = 40$).

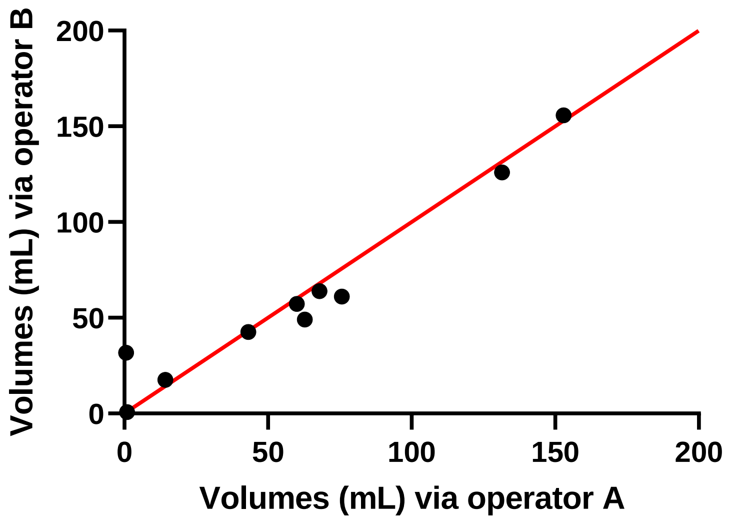

Figure S2. correlation of fluid volume in the total colon on the same datasets via the *2D*-protocol between operators A and B (Pearson's correlation coefficient 0.970, $p < 0.05$, $n = 10$).

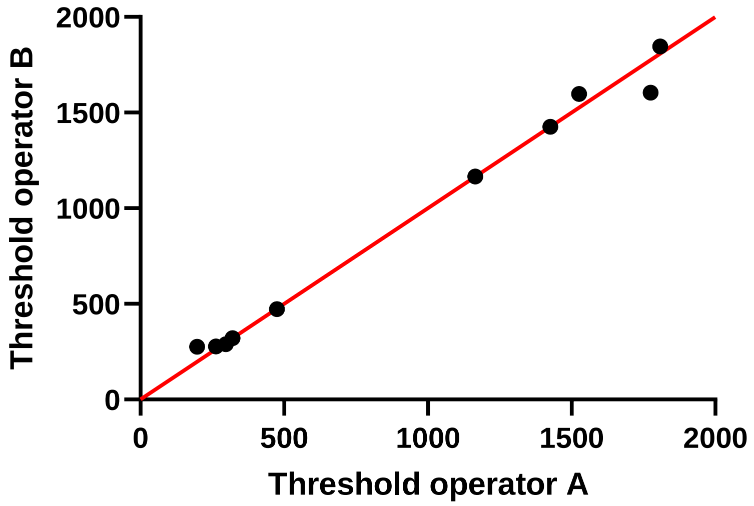

Figure S3. correlation between the CSF-treshold set on the same datasets between operators A and B (Pearson's correlation coefficient 0.995, $p < 0.05$, $n = 10$).
